# Performance of the TaqMan COVID-19 Pooling Kit for detection of SARS-CoV-2 in asymptomatic and symptomatic populations

**DOI:** 10.1371/journal.pone.0269798

**Published:** 2022-06-10

**Authors:** Troy Ganz, Sarah Sanderson, Connor Baush, Melanie Mejia, Manoj Gandhi, Jared Auclair

**Affiliations:** 1 Life Sciences Testing Center, Northeastern University, Burlington, MA, United States of America; 2 Thermo Fisher Scientific, South San Francisco, CA, United States of America; Regional Medical Research Centre Bhubaneswar, INDIA

## Abstract

Clinical evidence for asymptomatic cases of coronavirus disease (COVID-19) has reinforced the significance of effective surveillance testing programs. Quantitative reverse transcriptase polymerase chain reaction (RT-qPCR) assays are considered the ‘gold standard’ for detection of severe acute respiratory syndrome coronavirus 2 (SARS-CoV-2) RNA. However, the labor and resource requirements can be prohibitive with respect to large testing volumes associated with the pandemic. Pooled testing algorithms may serve to increase testing capacity with more efficient resource utilization. Due to the lack of carefully curated cohorts, there is limited evidence for the applicability of RT-PCR pooling in asymptomatic COVID-19 cases. In this study, we compared the analytical sensitivity of the TaqMan™ SARS-CoV-2 Pooling Assay to detect one positive sample in a pool of five anterior nares swabs in symptomatic and asymptomatic cohorts at an institute of higher education. Positive pools were deconvoluted and each individual sample was retested using the TaqPath™ COVID-19 Combo Kit. Both assays target the open reading frame (*ORF*) *1ab*, nucleocapsid (*N*), and spike (*S*) gene of the strain that originated in Wuhan, Hubei, China. Qualitative results demonstrated absolute agreement between pooled and deconvoluted samples in both cohorts. Independent t-test performed on C_t_ shifts supported an insignificant difference between cohorts with p-values of 0.306 (*Orf1ab*), 0.147 (*N*), and 0.052 (*S*). All negative pools were correctly reported as negative. Pooled PCR testing up to five samples is a valid method for surveillance testing of students and staff in a university setting, especially when the prevalence is expected to be low.

## Introduction

Efforts to mitigate the reproductive number (R_0_) for the coronavirus disease 2019 (COVID-19) pandemic, an emergent respiratory infection caused by severe acute respiratory syndrome coronavirus 2 (SARS-CoV-2), has been undermined by the potential for sustained transmission through pre-symptomatic, paucisymptomatic (subclinical), and asymptomatic carriers [1]. Growing evidence from immunological case studies and bioinformatic investigations have substantiated clinical diagnostic tests that have reported insignificant variance in results among patients that differ in symptomology. Asymptomatic patients have reportedly developed ground-glass opacities in lung tissue and significant adaptive immune (IgG and IgM) responses, however cell-mediated immune response from asymptomatic cases have shown to be less significant compared to symptomatic cases [2, 3]. A recent investigation noted a correlation between globally reported asymptomatic cases and a single nucleotide polymorphism (SNP) in SARS-CoV-2 RNA at position 11083G>T translating to a phenylalanine mutation (L37F) on non-structural protein 6 (*NSP6*) [4]. While a less severe immune response may be elicited in asymptomatic cases, the replication mechanisms in these individuals remain intact, and this necessitates effective risk management measures [5].

The analytical sensitivity of RT-qPCR has propelled its utility as the ‘gold standard’ to accurately detect SARS-CoV-2 RNA from upper respiratory specimens (e.g., nasopharyngeal, anterior nares, and saliva); however, the high-complexity format has merged with demands of a pandemic to generate severe shortages in materials and technical personnel. A resurgence of interest in group testing, or sample pooling, whereby the ratio of specimens to result is greater than one, has led investigators to optimize protocols that would allow an increase in testing capacity without requiring additional resources [6]. Caveats include a linear loss of sensitivity with sample dilution, and, in hierarchical systems, disease prevalence may determine efficiency as positive pools are deconvoluted to retest each patient individually [7]. Nevertheless, sample pooling stands to provide an accurate and affordable method for general population testing.

Since gaining USFDA emergency use authorization (EUA) for the TaqPath™ COVID-19 Combo Kit on March 24, 2020, Thermo Fisher Scientific, Inc. has developed a specialized RT-qPCR testing kit with enhanced sensitivity for the purposes of pooling up to five samples known as the TaqMan™ SARS-CoV-2 Pooling Assay. In the following study, we compared the sensitivity of the TaqMan™ assay to detect one positive sample in a pool of five from 30 symptomatic and 30 asymptomatic individuals that were previously characterized as positive using the TaqPath™ COVID-19 Combo Kit; 10 negative pools of five were included as a control group.

## Methods

### Sample collection, storage, and preanalytical processing

The study was conducted at the Northeastern University COVID-19 response laboratory, the Life Sciences Testing Center (LSTC; Burlington, MA, USA), which performs population testing for university affiliates. Students on campus are required to test every three days while staff and faculty on campus test twice a week [8]. Affiliates attending campus are required to complete an online daily wellness survey that identifies whether they have experienced COVID-19 related symptoms or have been in close contact with a positive COVID-19 case in the past 24 hours. Depending on the survey results, they are directed to get tested at either the non-symptomatic site (Cabot Center), where observed self-collection is performed, or the symptomatic site (Marino Center), where collection is performed by a healthcare professional. Temperature checks and general symptom screening were performed by trained personnel on the subjects at both sites to determine presence or absence of symptoms. Anterior nasal swabs (polyester) are collected in 3-mL BD Vacutainer® (cat: 366703; without additives, Mississauga, ON, Canada) tubes and transported dry to the LSTC facility [8]. Upon logging into the laboratory information system (Harvest™; Orchard Software Corporation, Carmel, IN), 3 mL of viral transport media (cat: VR2019-1L; Redoxica, Little Rock, AR) is added and the sample is shaken at 1 x g rpm for 5 minutes [8]. Processed specimens are stable for <72 hours at 2–8°C. Positive samples are catalogued, aliquoted, and stored indefinitely at -80°C. Depending on the site at which the samples are collected (Cabot or Marino), this monitoring approach allows for curated cohorts of symptomatic or asymptomatic cases.

### TaqMan™ SARS-CoV-2 Pooling assay: Pooled specimen RNA extraction and RT-qPCR setup

Pools consisting of five samples were constructed from one positive sample and four negative samples. Positive samples were selected at random from -80°C storage and thawed at 2–8°C. RNA extraction was performed using 400 μL of combined sample volume using the MagMAX™ Viral/Pathogen II (MVP II) Nucleic Acid Isolation Kit (cat: A48383; Thermo Fisher Scientific, Waltham, MA) by combining 10 μL of Proteinase K, 80 μL of each sample (400 μl pooled volume), 550 μL of lysis buffer with RNA binding beads, and 10 μL of MS2 phage extraction control to a single well of an Agilent 1 mL 96-well plate. The plate was vortexed 2 minutes at 3 x g, incubated 5 minutes at 65°C, vortexed for 5 minutes at 3 x g, and incubated at room temperature on a magnetic stand. After waste aspiration, the beads underwent three cycles of resuspension/aspiration in 1 mL wash buffer, 1 mL 80% ethanol, and 50 μL elution buffer, respectively. RT-qPCR was performed using 17.5 μL of eluant added to 7.5 μL of reaction mix that contained 6.25 μL TaqPath™ 1-Step RT-qPCR MM, CG (cat: A15299; Thermo Fisher, Waltham, MA) and 1.25 μL TaqMan™ SARS-CoV-2 Pooling Multiplex Assay (cat: A50790; Thermo Fisher, Waltham, MA).

### TaqPath™ COVID-19 Combo Kit: Deconvoluted specimen RNA extraction and RT-qPCR setup

Pools that tested positive were deconvoluted and each of the five component specimens were repeated using the TaqPath™ COVID-19 Combo Kit currently employed at the LSTC. As previously described [8], RNA extraction was performed using the MVPII kit with 5 μL of Proteinase K, 200 μL sample, 275 μL lysis buffer with RNA binding beads, and 5 μL MS2 phage extraction control. Purification procedure was performed using an Agilent Bravo liquid handler (Agilent Technologies, Santa Clara, CA) whereby, after initial shaking 2 minutes at 3 x g and 5 minutes incubation at 65°C, the beads underwent three cycles of resuspension/aspiration in 165 μL wash buffer, 165 μL 80% ethanol, and 50 μL elution buffer, respectively. RT-qPCR was performed using 10 μL of eluate added to 15 μL of reaction mix that contained 6.25 μL TaqPath™ 1-Step Multiplex Master Mix (cat: A28523; No ROX™, Thermo Fisher, Waltham, MA), 1.25 μL COVID-19 Real Time PCR Assay Multiplex (cat: A47814; Thermo Fisher, Waltham, MA), and 7.5 μL Nuclease-free water (cat: 4387936; Ambion™, Thermo Fisher, Waltham, MA).

### RT-qPCR thermal profile and results management

The thermal profile executed by Applied Biosystems™ 7500 Fast Dx Real-Time PCR Instrument (cat: 4406985; Thermo Fisher Scientific, Waltham, MA) was the same for both TaqMan™ and TaqPath™ methods and is described in detail in Ganz et al. [8]. Both assays contain primers and probes that target the SARS-CoV-2 open reading frame (*ORF*) 1ab, nucleocapsid (*N*), and spike (*S*) genes as well as the MS2 phage extraction control. Resulting threshold cycle (C_t_) values were extracted using Applied Biosystems COVID-19 Interpretive Software (version 1.5) and analyzed using GraphPad Prism version 9.3.0 (GraphPad Software, La Jolla, CA) and R version 4.2.0 (R Core Team (2022), Vienna, AUT). Qualitative endpoints set to determine target presence were C_t_≤37 for SARS-CoV-2 genes and C_t_≤32 for MS2 control. Determinations were ruled as positive if two or more SARS-CoV-2 genes were detected, and indeterminant if one SARS-CoV-2 genes was detected.

## Results and discussion

**Fig 1:**
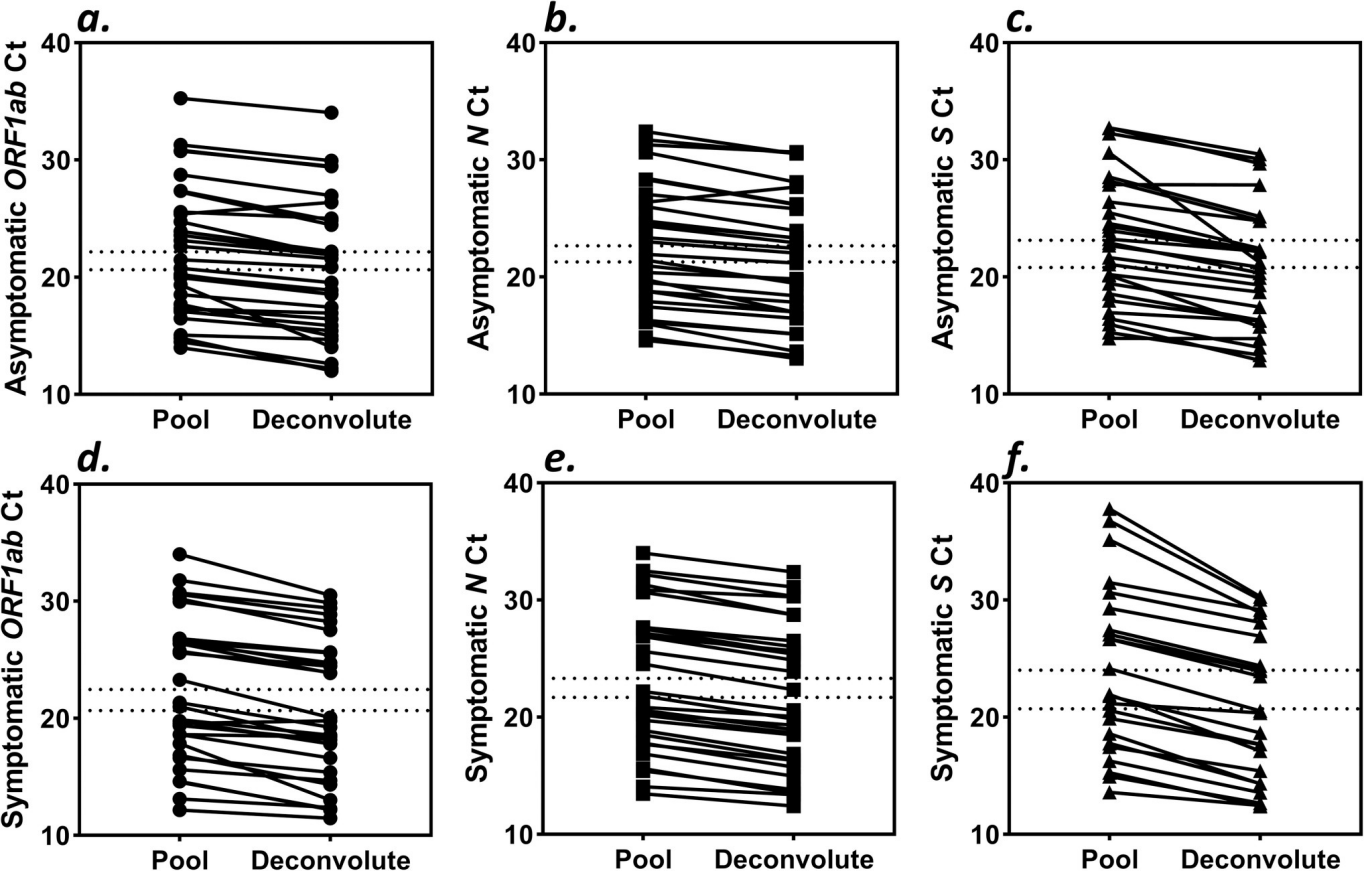
Trend of C_t_ shift from pooled and deconvoluted result. Pairwise comparisons of pooled and deconvoluted samples for each SARS-CoV-2 gene target from Asymptomatic (*a*,*b*,*c*) and Symptomatic cohorts (*d*,*e*,*f*); ORF1ab, circles; N gene, squares; S gene, triangles; mean C_t_ values, dashed intersects.

**Fig 2:**
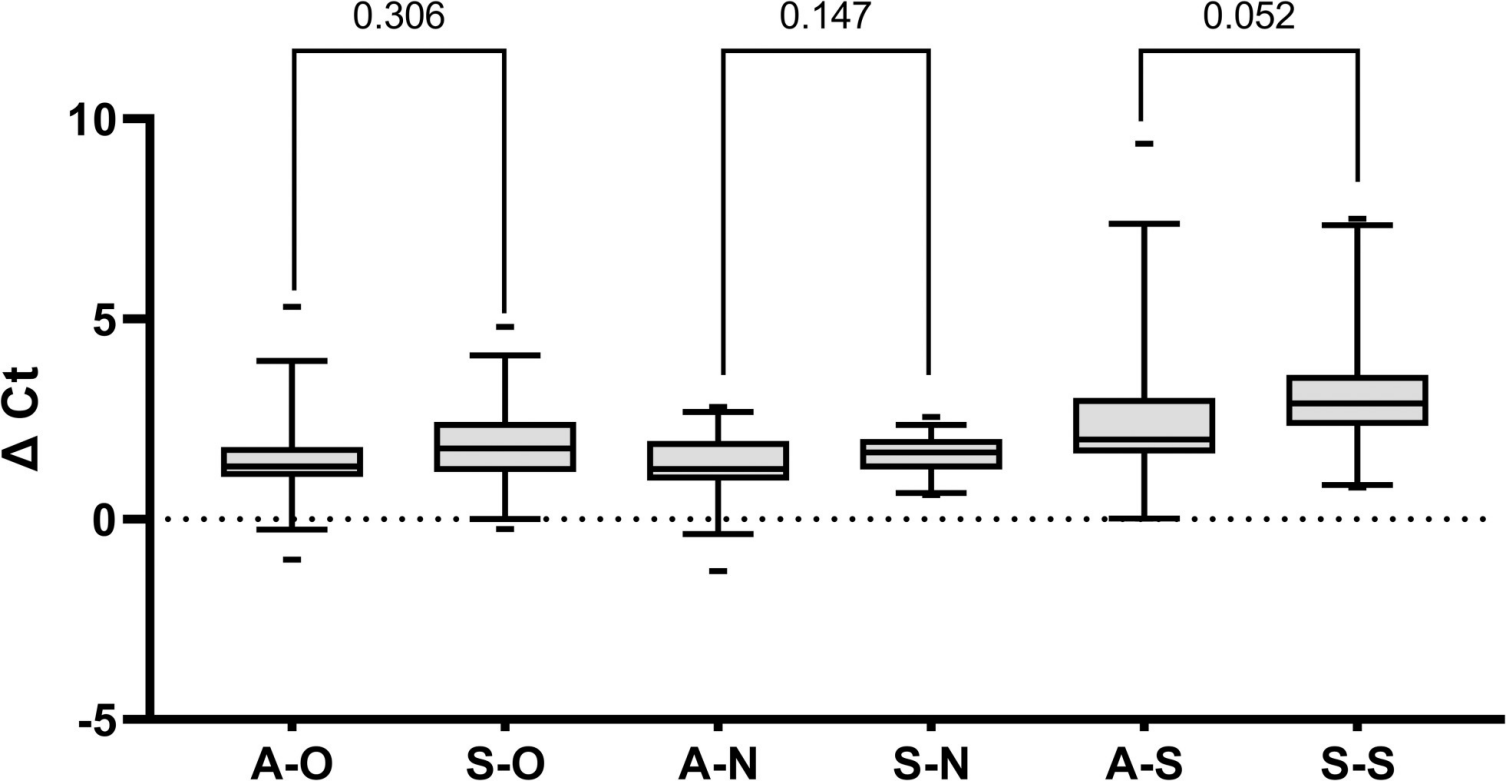
Asymptomatic and symptomatic C_t_ shift. Box-and-whisker plot describing C_t_ shift (ΔC_t_) for each gene from each cohort; whiskers, 5–95 percentiles; min-max range, dash mark; p-values annotated; A, Asymptomatic; S, Symptomatic; O, *ORF1ab* gene; N, *N* gene; S, *S* gene.

Average monthly prevalence estimates ranging August 2020 to April 2021 for the Cabot Center (non-symptomatic) was 0.16% (95% CI 0.07%-0.25%) and Marino Center (symptomatic) was 3.33% (95% CI 1.73%-4.93%). Thirty samples from each of the two sites were used to generate 60 five-sample pools with 240 negative samples. The TaqMan™ pooling method detected SARS-CoV-2 in each of the 60 five-sample pools and statistical analysis was performed using the deconvoluted result. Average C_t_ values for the pooled asymptomatic samples were 22.17 (95% CI 20.06–24.28), 22.66 (95% CI 20.63–24.69), and 23.15 (95% CI 20.99–25.31) and the deconvoluted asymptomatic samples were 20.64 (95% CI 18.47–22.80), 21.30 (95% CI 19.25–23.34), and 20.80 (95% CI 18.79–22.82) for the *ORF1ab*, *N*, and *S* genes, respectively (Fig 1A–1C). Average C_t_ values for the pooled symptomatic samples were 22.47 (95% CI 20.18–24.76), 23.31 (95% CI 21.06–25.57), and 24.01 (95% CI 20.92–27.10) and the deconvoluted symptomatic samples were 20.65 (95% CI 18.40–22.90), 21.70 (95% CI 19.47–23.93), and 21.17 (95% CI 18.44–23.91) for the *ORF1ab*, *N*, and *S* genes, respectively (Fig 1D–1F). Average C_t_ shifts of 1.54±1.09 (*ORF1ab*), 1.37±0.79 (*N*), and 2.34±1.71 (*S*) for the asymptomatic cohort and 1.82±1.03 (*ORF1ab*), 1.62±0.48 (*N*), and 3.29±1.65 (*S*) for the symptomatic cohort were observed. In efficient PCR amplification, a 2-fold dilution will result in shift of 1 C_t_ value. To determine statistical significance of C_t_ shift between pooled and deconvoluted result, a permutation test of 10^6^ iterations based on the difference of means generated p-values of 0.152 (*ORF1ab)*, 0.167 (*N*), and 0.055 (*S)* for asymptomatic samples and 0.125 (*ORF1ab*), 0.149 (*N)*, and 0.082 (*S)* for symptomatic samples. Additionally, independent t-test did not confirm a statistical significance in C_t_ shift between the asymptomatic and symptomatic cohorts with p-values of 0.306, 0.147, and 0.052 for *ORF1ab*, *N*, and *S* genes, respectively (Fig 2). Each negative pool reported negative and was not deconvoluted. A level of significance of α = 0.05 was used.

This study sought to confirm SARS-CoV-2 infections that cause mild immune responses proliferate to similar extents as those that elicit severe cases of COVID-19, which enables equal probability of detection in RT-qPCR assay in pools of five with minimal loss of sensitivity. At the time of this study (April 2021), prevalence of SARS-CoV-2 variant B.1.1.7, apparent by S gene target failure, had increased by an order of magnitude since November 2020 and represented 7% of asymptomatic and 20% of symptomatic cases of the respective sample sizes [9]. The novel strategy employed by the TaqMan™ COVID-19 Pooling Kit method involved increased extraction and elution volumes for group tests that resulted in absolute qualitative agreement to the deconvoluted counterpart, including a confirmed indeterminant case, with a more conserved C_t_ shift [7, 8, 10]. Since symptomatic subjects tend to have a significantly higher prevalence of COVID-19 in a pandemic setting, from a resource efficiency standpoint, it is advisable to consider pooling samples when testing subjects who are non-symptomatic such as in vaccinated populations, where the prevalence rates are expected to be significantly lower.

### Dual publication

The LSTC has recently published an evaluation of sample pooling using the TaqPath™ COVID-19 Combo Kit (Thermo Fisher, Waltham, MA) in Ganz TJ, Donner R, Hines KM, Waithe-Alleyne ML, Slate DL, Abel G, Auclair JR. Two-Stage Hierarchical Group Testing Strategy to Increase SARS-CoV-2 Testing Capacity at an Institution of Higher Education: A Retrospective Analysis. 2021. The Journal of Molecular Diagnostics. Only sections of the methods demonstrate similarity (e.g., ‘Sample Collection, Storage, and Pre-Analytical Processing’, ‘TaqPath™ COVID-19 Combo Kit: Deconvoluted Specimen RNA Extraction and RT-qPCR Setup’, and ‘RT-qPCR Thermal Profile and Results Management’) as such information was relevant to the work and necessary to maintain.
